# COVID-19 vaccination rate in patients with mental illness in a psychiatric hospital: a cohort study

**DOI:** 10.1192/j.eurpsy.2023.1654

**Published:** 2023-07-19

**Authors:** D. Areias, C. Portela, R. Dionísio, S. M. Sousa, E. G. Pereira

**Affiliations:** Psychiatry Inpatient Unit, Hospital Magalhães Lemos, Porto, Portugal

## Abstract

**Introduction:**

Individuals with severe mental health problems are at greater risk of COVID-19 infection and increased risk of hospitalization and mortality. Vaccination against COVID-19 has demonstrated its importance in preventing and reducing these negative outcomes.

**Objectives:**

This study aims to assess the vaccination rate of people with mental illness in comparison with the general population.

**Methods:**

We will conduct a retrospective evaluation of vaccine uptake in a sample of patients admitted to a psychiatric hospital between the 1^st^ of July of 2021 and the 30^th^ of June of 2022 in the Porto region. According to their vaccination plan, all patients were offered the possibility vaccination. Statistical analysis will be performed to analyse the data.

**Results:**

We expect to assess over 1500 patients. Regarding other studies on the same subject, although in different countries, we may predict that the vaccination rate in our sample will not, statistically, differ from the general population.

**Conclusions:**

Some studies have shown higher resistance and hesitancy towards the COVID-19 vaccination in mental health patients, however others did not find differences between these patients and the general population. Therefore, this study will allow us to better understand the impact of mental illness in the vaccination rate in our population.

**Disclosure of Interest:**

None Declared

